# The Effects of HIV Infection on the Immune Response to Malaria Among Pregnant Women in Kumba, Southwest Cameroon: Protocol for a Cross-sectional Study

**DOI:** 10.2196/38213

**Published:** 2023-01-24

**Authors:** Bekindaka Ngemani Obase, Zeukeng Francis, Esemu Livo Forgu, Awanakam Honore, Jude Daiga Bigoga, Dickson S Nsagha

**Affiliations:** 1 Pan African University of Life and Earth Science Institute (Including Health and Agriculture) University of Ibadan Ibadan Nigeria; 2 College of Medicine University of Ibadan Teaching Hospital Ibadan Nigeria; 3 The Biotechnology Center University of Yaoundé I Yaoundé Cameroon; 4 Department of Biochemistry and Biotechnology Faculty of Science University of Buea Buea Cameroon; 5 Centre for Research on Emerging and Reemerging Diseases Institute of Medical Research and Medicinal Plant Studies Yaoundé Cameroon; 6 Department of Public Health and Hygiene Faculty of Health Sciences University of Buea Buea Cameroon

**Keywords:** malaria, HIV, co-infection, pregnancy, prevalence, allele, immunity, antibodies, cytokines, Cameroon

## Abstract

**Background:**

Malaria and HIV, 2 of the world's deadliest diseases, share a lot of territory in sub-Saharan Africa.

**Objective:**

This study seeks to investigate the effect of HIV on the immune response to malaria infection among pregnant women in Kumba in the southwest region (SWR) of Cameroon. The study aims to determine the prevalence of malaria infection, assess the occurrence of Plasmodium falciparum genetic diversity, and evaluate the antibody (immunoglobulin [Ig]G and IgM: apical membrane antigen-1 [AMA1], merozoite surface protein [MSP]1, MSP2, MSP3, and erythrocyte-binding antigen [EBA]175) and cytokine (interleukin [IL]-10, tumor necrosis factor alpha [TNF-α], and interferon gamma [IFNγ]) response to malaria infection among pregnant women with and without HIV in Kumba.

**Methods:**

The study will be a hospital-based cross-sectional design that will run from March 2022 to February 2023. It will recruit pregnant women with and without HIV who are in their third trimester of pregnancy. The study will be carried out in 5 health institutions in Kumba: General Hospital Kumba, Presbyterian Hospital Kumba, District Hospital Kumba-town, Kossala Integrated Health Center Kumba, and Catholic Hospital Kumba. About 3 mL of the mother’s venous blood, placental blood, and baby cord blood will be collected from each pregnant women at the point of delivery. Microscopy, rapid diagnostic tests (RDTs), and nested polymerase chain reaction (PCR) will be performed to identify the malaria parasite in all the samples, and nested PCR targeting the different genetic diversity markers for *P. falciparum* will also be performed. Furthermore, sequencing will be performed to study the nucleotide sequence of different alleles, and the genetic diversity of the alleles responsible for malaria infection among pregnant women will be assessed. A multiplex assay will be conducted to analyze the peripheral blood plasma and cord blood plasma for the cytokine and total antibody response to malaria infection among pregnant women with and without HIV. The questionnaire for data collection will be pretested at the Kumba District Hospital, and ethical clearance will be obtained from the University of Buea and the Regional Delegation of Public Health for the SWR. Data will be analyzed using SPSS Statistics and STATA. All *P* values <.05 will be considered statistically significant. BioEdit 7.0.0 software will be used to align the nucleotide sequences of different genes after sequencing. Phylogenetic tree searching will be conducted using the maximum-likelihood (ML) method in MEGA V6.0.

**Results:**

The project started in March 2022 and will end in February 2023. Presently, three-fourth of the project funding has been disbursed to date. A total of 218 participants have been enrolled: 193 (88.5%) women without HIV and 25 (11.5%) women with HIV. Between February 2023 and March 2024, the following results will be ready for publication: maternal-neonatal malaria prevalence among pregnant women and babies in Kumba, the effect of HIV on (1) *P. falciparum* genetic diversity among pregnant women in Kumba, (2) the maternal and neonatal immune response to MSP1, MSP2, and EBA175 IgG antibody response to *P. falciparum*–caused malaria infection among pregnant women, and (3) the maternal and neonatal pro-inflammatory and anti-inflammatory cytokine response to malaria infection.

**Conclusions:**

HIV infection increases the prevalence of malaria infection among pregnant women and also influences the genetic diversity of *P. falciparum*, with MSP1 alleles being the most prevalent. HIV infection also reduces the antibody response to malaria infection, as well as altering the level of pro-inflammatory and anti-inflammatory responses to malaria infection.

**International Registered Report Identifier (IRRID):**

DERR1-10.2196/38213

## Introduction

Malaria and HIV, 2 of the world's deadliest diseases, share a lot of territory in sub-Saharan Africa. As a result, malaria and HIV coinfection is common in the region [1]. In 2019, malaria alone affected over 229 million people worldwide, with the majority (95%) of the cases occurring in 29 countries in sub-Saharan Africa: Nigeria (27%), Democratic Republic of Congo (12%), Uganda (5%), Mozambique (4%), and Niger (3%) [2]. Infection with malaria in humans is caused by 6 species, with *Plasmodium falciparum* being the most virulent of them all [3]. In Cameroon, malaria is a significant public health issue, with the whole nation in danger of transmission [4,5]. Despite considerable progress in recent years to reduce the burden of this infection due to the implementation of several malaria control strategic programs, the disease remains widely spread, with an estimated 3.3-3.7 million probable cases in health care facilities each year [4,6].

Infection with HIV or malaria during pregnancy is often harmful to both mother and child [7]. Coinfection raises the chances of adverse outcomes, which include maternal anemia, increased parasite sequestration at the placenta, low-birth-weight babies, and immune dysregulation to malaria [7]. The immunological reasons behind the increased malaria sensitivity of women with HIV, as well as the effect of coinfection on HIV transmission from mother to child, are major public health problems. Once again, these increased risks are greatest in multigravidae [8]. Epidemiological studies suggest that pregnant women with HIV have a considerable decrease in the acquired immunity to malaria, resulting in a diminished ability to limit *P. falciparum* infection. This finding is corroborated by the fact that sulfadoxine-pyrimethamine–intermittent preventive treatment's (SP-IPT) effectiveness in pregnant women with HIV reduces [7,8].

Prior studies have shown that HIV infection interferes with the breadth of the antibody response to merozoite surface protein (MSP) antigens. According to Daniel et al [9], the antibody response to MSP1, MSP2, and apical membrane antigen-1 (AMA1) reduced in children with HIV, and only AMA1 was statistically significant [9]. HIV was associated with a restricted spectrum of responses to certain merozoite antigens. HIV changed the pace of age-related antibody acquisition against schizont extract but not the rate of age-related response formation to particular merozoite antigens [9]. Another research in Kenya found that serum antibody levels to AMA1 and (MSP1 block 2, MSP2, and MSP3) are negatively associated with the likelihood of acquiring malaria, whereas levels of MSP1 and erythrocyte-binding antigen (EBA)175 are not [10]. Although the findings of this research are interesting, they are inconsistent. As a result, additional research is necessary.

Among pregnant women, studies have shown that HIV dysregulates the immune response to malaria infection, making them more vulnerable to malaria [11]. HIV decreases immunoglobulin (Ig)G levels to circumsporozoite protein (CSP) and terminal complement complex (TCC) in women with infection, while cord IgG to CSP, MSP1, and TCC is considerably lower in neonates delivered to mothers with HIV [12]. This study found that HIV infection is related to decreased levels of cord antibodies to malarial antigens and that hypergammaglobulinemia may contribute to impaired antibody transmission [12]. Another study found that interferon gamma (IFNγ) and tumor necrotic factor alpha (TNF-α) levels are lower in pregnant women who have both malaria and HIV infections. Nonetheless, pregnant women with coinfection had higher mean plasma TNF-α concentrations. It was also shown that interleukin-2 (IL-2) levels decreased in mothers with HIV and with coinfection before increasing in mothers with malaria. IL-10 levels among women with coinfection also decreased when compared to women without infection. There were strong significant associations between plasma IL-2 levels and infection status [13].

Djontu et al [14] revealed that placental malaria reduces maternal plasma levels of cytokines IL-17E, IL-27, and IL-28A, as well as neonatal plasma levels of cytokines IL-17E and IL-28A, which may aid in parasite clearance and enhance infant birth weight. IL-27 and IL-6 are 2 cytokines that can have both pro-inflammatory and anti-inflammatory actions. Indeed, IL-27 has been shown to activate the T-bet transcription factor, which promotes helper T (Th)1 cell differentiation [15,16]. Other investigations have shown that it suppresses the growth of IL-17–producing Th17 cells [17] and decreases the Th2 response [17]. IL-6 suppresses transforming growth factor (TGF)–induced IL-10–producing regulatory T-cell (Treg) development [18], but when combined with TGF-α, it preferentially promotes IL-17–producing Th17 cell differentiation [19]. Mucosal epithelial cells, as well as several immune cell types, such as mast cells, macrophages, eosinophils, Th2 cells, and NKT cells, can generate IL-17E (IL-25) [20,21]. It promotes Th2 cell responses, while suppressing Th17 cell responses [22]. Previous research identified IL-28A as a newly defined type III IFN, with antiviral properties [22,23] and generated primarily by antigen-presenting cells (APCs) [23]. This cytokine can enhance Th17-induced inflammation, offer Th1 polarization, and suppress Th2 responses [24]. As a result, IL-6, IL-27, IL-17E, and IL-28A are key cytokines that may be implicated in the control of human immunological disorders caused by Th1, Th2, Treg, and Th-17 response imbalance [25].

Cameroon approved the option B+ treatment strategy 2014 to prevent mother-to-child transmission (MTCT) of HIV in 2014 [24,26]. However, effevirenz has lately been replaced with dolutegravir. According to data of a trial study conducted in Cameroon, a dolutegravir-based regimen, which is a low-cost, generic, fixed-dose antiretroviral therapy (ART) combination containing tenofovir, lamivudine, and dolutegravir (TLD), is the preferred first-line treatment for patients with HIV-1 infection [25]. Nevertheless, there are concerns about the overall risks and benefits of use in women of childbearing potential [27]. That is why this study aims to determine the prevalence of malaria infection, assess the occurrence of *P. falciparum* genetic diversity, and evaluate the antibody (IgG and IgM: AMA1, MSP1, MSP2, MSP3, and EBA175) and cytokine (IL-10, TNF-α, and IFNγ) response to malaria infection among pregnant women with and without HIV in Kumba.

## Methods

### Study Design and Population

This will be a hospital-based cross-sectional study that will run from March 2022 to February 2023. It will enroll pregnant women with and without HIV who are in the third trimester of pregnancy (26-32 weeks) and have willingly accepted to participate in the study by signing a consent form. During delivery, samples will be collected and questionnaires will be appropriately filled.

### Study Site

The study will be conducted in Kumba, which is a metropolitan city in Meme Division of the southwest region (SWR) of Western Cameroon. Meme Division is 1 of the 6 divisions of the SWR of Cameroon. It is made up of 5 subdivisions: Kumba I, II, and III (largely urban) and Mbonge and Konye (dominantly rural) [28].

### Ethical Considerations

Ethical clearance for this study has been obtained from the Institute for Advanced Medical Research and Training (IAMRAT), College of Medicine, University of Ibadan, Nigeria (ID NHREC/05/01/2008a). However, ethical clearance from Cameroon has been obtained from the Institutional Review Board of the Faculty of Health Sciences at the University of Buea (ID 2022/1655-02/UB/SG/IRB/FHS). Administrative clearance has been obtained from the Ministry of Public Health, Regional Delegation of Public Health for the SWR (ID R11 /MINSANTE/SWR/RDPH/PS/276/281). Further authorization has been obtained from the Meme Health District Health service (ID 12/022/MINSANTE/RDPHSW/KHD/DMO/048). Directors of the health facilities involved have also provided an authorization letter for the study to take place in their various facilities. Participants who wish to participate in the study will first sign an informed consent form. For participants who are unable to read or write, the information will be read out and explained to them and their thumbprints will be taken. The study will consider pregnant women with and without HIV who are in the third trimester of pregnancy and are fully aware of their viral load counts.

### Sample Size Determination

The estimated sample size for the study was calculated using the Cochrane formula [29]. The estimated prevalence of malaria among pregnant women with HIV is 81%, as obtained from a study looking at malaria prevalence in pregnant women with and without HIV in neighboring Nigeria [30]. The critical value and standard value for the corresponding level of confidence were 1.96, and the margin of error was 0.05. The estimated sample size is 236. The ratio of pregnant women with to those without HIV will be 1:1, which therefore implies that the total estimated sample size is 472. That is, 236 (50%) pregnant women with and 236 (50%) pregnant women without HIV will be enrolled in the study.

### Inclusion and Exclusion Criteria

#### Inclusion Criteria

The study will enroll only pregnant women who are in their third trimester of pregnancy—that is, within 26-32 weeks of pregnancy—and who willingly provide their consent to participate in the study. The study will also consider only pregnant women who are aware of their HIV status as well as viral load. In addition, the study will accept only those attending antenatal care (ANC) visits at the selected health facilities and who are willing to deliver in the facility of enrolment.

#### Exclusion Criteria

The study will not consider pregnant women who are not in their third trimester of pregnancy and have not signed the informed consent form to participate in the study. All those who will be attending ANC visits at the selected health facilities but are not willing to deliver in those health facilities will not be considered.

### Sampling Technique

A probability proportionate to size sampling will be used to determine the number of participants to be recruited in each health institution (Table 1). A simple random sampling method will be used each day to recruit participants as they come for their ANC visits to the selected health facilities.

**Table 1:** Number of participants to be recruited in each of the selected health facilities (N=1180).

Sample	General Hospital Kumba				District Hospital Kumba-town				Presbyterian Hospital Kumba				Kossala Integrated Health Center Kumba				Catholic Hospital Kumba		
	2021 Stat^a^, n (%)	*P* value	PSS^b^, n (%)	2021 Stat, n (%)		*P* value	PSS, n (%)	2021 Stat, n (%)		*P* value	PSS, n (%)	2021 Stat, n (%)		*P* value	PSS, n (%)	2021 Stat, n (%)		*P* value	PSS, n (%)
Pregnant women with HIV	10/718 (1.4)	.014	3/27 (11.1)	10/280 (3.6)		0.036	8/27 (29.6)	20/800 (2.5)		.025	6/27 (22.2)	13/765 (1.7)		.016	4/27 (14.8)	17/685 (2.5)		.025	6/27 (22.2)
Pregnant women without HIV	708/718 (98.6)	.986	233/1153 (20.2)	270/280 (96.4)		0.964	228/1153 (19.8)	780 /800 (97.5)		.975	230/1153 (19.9)	752/765 (98.3)		.983	232/1153 (20.1)	668/685 (97.5)		.975	230/1153 (19.9)

^a^Stat: statistics.

^b^PSS: probability sample size.

### Sociodemographic and Clinical Baseline of Study Participants

A well-structured semiquestionnaire will be used to collect demographic and clinical parameters from study participants. The questionnaire will be pretested at District Hospital Kumba-town before being issued to participants for data collection.

The result of the rapid diagnostic test and microscopy for each participant will be made available to them. This is to ensure that those positive for malaria should be placed immediately on treatment.

### Sample Collection

Briefly, 3 mL of the mother’s peripheral (venous) blood, placental blood, and baby cord blood will be collected into EDTA, heparinized, and sodium-citrated tubes, respectively. A capillary puncture will be made to collect the sample, and a thick-film smear will be prepared for microscopy. Smears will be allowed to air-dry and then carefully placed in well-cleaned slide boxes to be stored for further analysis.

### Laboratory Analyses

#### Thick-Film Microscopy

The thick-film smears will be stained with 10% Giemsa for 15 minutes, and the slides will be rinsed and allowed to air-dry. After that, the smears will be examined for the presence of *P. falciparum* under the 100X oil emission objective of the microscope. The parasite density will be calculated using the formula [31]:

Parasite density = (Number of parasites counted per 200 white blood cells [WBCs]/200) × 8000.

#### Rapid Diagnostic Test for Malaria

A drop of about 50 µL will be used to determine malaria infection in all the enrolled participants using an SD-Bioline RDT kit, which detects histidine-rich protein II and lactate dehydrogenase. After the application of the sample and buffer, the kit will be allowed to stand for 15 minutes according to the manufacturer’s instructions, and results will be interpreted qualitatively and recorded.

#### DNA Extraction

Drops of venous, placental, and cord blood will be blotted on filter paper and allowed to air-dry. The blotted filter paper will be used to extract the DNA of malaria parasites using the hot chelex extraction method, as described by Musapa et al [32].

#### Nested Polymerase Chain Reaction

The DNA extract will be used to perform nested PCR using the mitochondria (cytochrome C oxidase 3 [Cox-3]) primers for the detection of *P. falciparum,* as described by Lloyd et al [33].

#### Genetic Diversity of MSP1, MSP2, and EBA175 Using Nested PCR

A portion of the DNA extract from placental and cord blood will be used to determine the genetic diversity for samples that test positive for *P. falciparum* at the level of placental and cord blood. The 3 polymorphism loci MSP1, MSP2, and EBA175 will be amplified using specific primers targeting the alleles of *P. falciparum* in nested PCR. Primary amplification followed by secondary PCR using specific primers for K1, Mad20, and RO33 (for *MSP1*) and 3D7 and FC27 (for *MSP2*) allelic families will be performed [34]. Primer sequences have been previously described [35]. For the EBA175 loci, specific primers targeting the F1 loop, F2 loop, and C loop will be used to determine the different alleles responsible for infection, as described previously [36].

#### Sequencing Analysis

A total of 10 samples positive for *MSP1*, *MSP2*, *MSP3*, and *EBA175* at the level of placental and cord blood will be randomly selected and sent for analysis to the Inqaba Biotechnical Industry in Nigeria for sequencing to identify and study the DNA sequences of the alleles responsible for placental and transplacental infection.

#### Cytokine and Antibody Analysis Using the Luminex Technique

**Figure 1:**
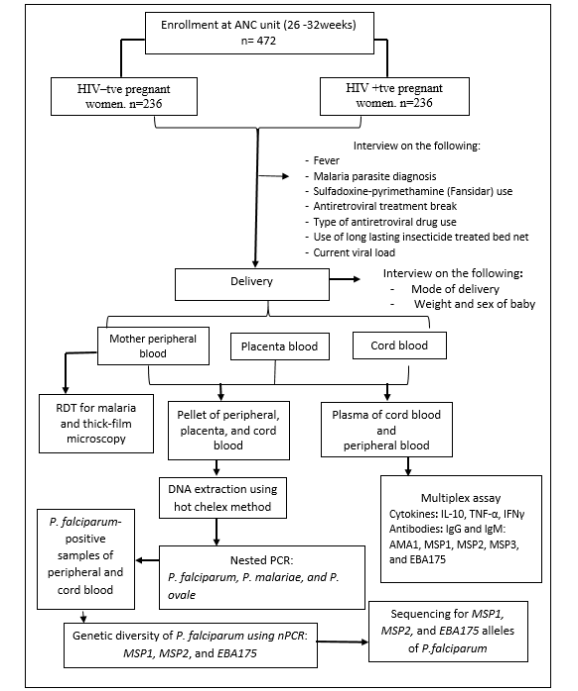
Summary flowchart for the recruitment of participants into the study. AMA1: apical membrane antigen-1; ANC: antenatal care; EBA: erythrocyte-binding antigen; IFNγ: interferon gamma; IgG: immunoglobulin; IgM: apical membrane antigen-1; IL: interleukin; MSP: merozoite surface protein; nPCR: nested polymerase chain reaction; PCR: polymerase chain reaction; *P. falciparum*: *Plasmodium falciparum*; *P. malariae: Plasmodium malariae*; *P. ovale: Plasmodium ovale*; RDT: rapid diagnostic test; TNF-α: tumor necrosis factor alpha.

The stored plasma will be used to determine levels of cytokines IL-10, TNF-α, and IFNγ using a magnetic multiplex screening assay (Luminex) with the help of a Human Multi-Analyte Premixed Kit (R&D Systems Inc, Minneapolis, MN, USA) [14]. A portion of the plasma will be used to evaluate the IgG antibody response level to MSP1, MSP2, and EBA175 using the multiplex assay, as described in previous studies for the detection of IgG antibodies to malaria [12,37]. A summary flowchart of the entire study can be seen in Figure 1.

### Data Management and Analyses

Data will be entered into Microsoft Excel, cleaned, and transformed for the different analyses to be performed. SPSS Statistics (IBM Corp) and STATA will be used for statistical analysis. A normality test will be performed to determine the different parametric and nonparametric tests to be used for the different analyses.

Descriptive analysis of the general study population will be conducted, and both the mean and median of each parameter will be considered. The chi-square test will be performed to analyze categorical variables, and the Student *t*-test/Mann-Whitney *U* test will be performed to analyze continuous and categorical variables. The Kruskal-Wallis test/ANOVA will be conducted to analyze continuous and more than 2 categorical variables, while linear regression/Spearman correlation will be performed for multivariate analysis to determine the strength of association between different factors of the study and our dependent variables. All *P* values <.05 will be considered statistically significant. BioEdit 7.0.0 software will be used to align the nucleotide sequences of different genes after sequencing. Phylogenetic tree searching will be performed using the maximum-likelihood (ML) method in MEGA V6.0.

## Results

The prevalence of malaria infection; the occurrence of *P. falciparum* genetic diversity of MSP1, MSP2, MSP3, and EBA175; and the total antibody (IgG and IgM: AMA1, MSP1, MSP2, MSP3, and EBA175) and cytokine (IL-10, TNF-α, and IFNγ) response to malaria among pregnant women with and without HIV will be evaluated.

The first part of the project funding was in December 2021, the second part was in May 2022, and the third part was in December 2022. Currently, three-fourth of the project funds have been released. Data and sample collection started in March 2022 and will end in February 2023. A total of 218 participants have been enrolled: 193 (88.5%) pregnant women without HIV and 25 (11.5%) pregnant women with HIV (see Table 2).

**Table 2:** Current level of enrollment of participants into the study (N=218).

Sample	General Hospital Kumba, n (%)	District Hospital Kumba-town, n (%)	Presbyterian Hospital Kumba, n (%)	Kossala Integrated Health Center Kumba, n (%)	Catholic Hospital Kumba, n (%)	Total, n (%)
Pregnant women with HIV (n=25)	7 (28)	6 (24)	6 (24)	0	6 (24)	25 (100)
Pregnant women without HIV (n=193)	25 (13.0)	40 (20.7)	10 (5.2)	33 (17.1)	85 (44.0)	193 (100)
Total	31 (14.2)	45 (20.6)	16 (7.3)	33 (15.1)	93 (42.6)	218 (100)

## Discussion

### Main Findings

Immune development during pregnancy is important as it determines the resistance of pregnant women to malaria transmission. Few studies have shown that women develop protective immunity against pregnancy-associated malaria (PAM), which is thought to be due to the acquisition of antibodies to the parasite variant surface antigen 2 chondroitin sulphate A (VAR2CSA) [38]. This immunity becomes stronger, especially in multigravidae [38]. However, this does not appear to be the case with pregnant women with HIV, as studies have shown that HIV alters the immunity against malaria, making both primigravidae and multigravidae vulnerable to PAM [11]. Although studies looking at malaria prevalence have been carried out in Kumba, studies evaluating the immune response of pregnant women with and without HIV to malaria have not been conducted. This study will attempt to determine the prevalence of malaria infection, assess the occurrence of *P. falciparum* genetic diversity, and evaluate the antibody (IgG and IgM: AMA1, MSP1, MSP2, MSP3, and EBA175) and cytokine (IL-10, TNF-α, and IFNγ) response to malaria infection among pregnant women with and without HIV in Kumba.

In Nigeria, the prevalence of malaria infection among pregnant mothers with HIV was 81% compared to 75% among pregnant women without HIV [30]. When compared to mothers without HIV, mothers with HIV have more moderate parasitemia [30]. This finding is in line with a similar study from Kumba, Cameroon, which reported a 61.5% prevalence of malaria among pregnant women with HIV [39]. Even though the prevalence of malaria in Kumba is high, it would have been much higher if more sensitive diagnostic techniques, such as PCR, had been used for diagnosis in this study rather than traditional microscopy, whose sensitivity is dependent on technician expertise [40], and RDTs, which lack specificity due to the high possibility of cross-reaction of antibodies [39]. Kenji et al [40] discovered that PCR could diagnose 64 misdiagnosed cases that were missed by RDTs and microscopy. In addition, in the Republic of Congo, it was discovered that the prevalence of malaria infection is high in pregnant women attending antenatal facilities or hospitalized and that it increases when HIV infection is present [41]. However, in Jos, Nigeria, the prevalence of malaria infection reduced from 34.1% to 7.2% among women administered cotrimozaxole and from 25% to 10% among women administered SP-IPT for malaria prophylaxis [42]. According to some studies, HIV and AIDS cause immunosuppression, which reduces the overall immune response to malaria parasitemia, increasing the frequency of clinical malaria attacks [43,44]. Effective implementation of IPT strategies among pregnant women reduces their likelihood of malaria infection [42]. Nevertheless, studies in Ghana have also shown that HIV infection increases the rate of vertical transmission of malaria to children born to a mother with HIV [45]. This study was able to show that HIV-malaria coinfection is a significant predictor of adverse perinatal outcomes and neonatal morbidity [45]. However, although national guidelines in Cameroon recommend the use of cotrimozaxole for pregnant women with HIV and SP for pregnant women without HIV at all ANC visits [46], most health facilities suffer from drug shortages, forcing women to attend some ANC visits without medication. The effect can be seen in a study carried out in Cameroon, where not taking Fansidar for IPT was found to be associated with the development of malaria among pregnant women [1].

Studies have shown that *P. falciparum*, the most virulent species, has a wide genetic variety at all degrees of endemicity, which is not surprising, given that genetic diversity has been shown to be a function of transmission in specific regions [47,48]. Oboh et al [49] discovered that the prevalence of the *FC27* allele type in pregnant women isolates is higher (33.3%) than that in nonpregnant women (29.2%) when looking at the genetic diversity of *P. falciparum* (*MSP1* and *MSP2*) infection among pregnant women in Lagos, Southwest Nigeria [49]. This finding is in line with a similar study carried out in Yaoundé, Cameroon [50]. Although no study has been conducted to assess the diversity of *P. falciparum* among pregnant women with and without HIV as 2 distinct entities, a couple of studies have evaluated the diversity of *P. falciparum* among individuals with HIV. In Mutengene, Cameroon, it was reported that the MSP1 allelic variants detected among patients with HIV were KI (65.22%), MAD20 (27.54%), and RO33 (27.54%), while the MSP2 allelic variants were FC27 (30.43%) and 3D7/IC (31.88%), which was high and associated with anemia [51]. A study in Senegal reported no difference in genetic diversity between *P. falciparum* isolates from patients with malaria and concurrent malaria-arbovirus in Kedougou [52]. However, the increased number of genotypes observed in the malaria group was attributed to the larger number of samples rather than the extensive genetic diversity of *P. falciparum* isolates from patients with malaria [52]. The frequency of all MSP1 allelic families (K1, MAD20, and RO33) and the IC/3D7 allelic family of MSP2 was high (>70%) and comparable between the 2 study groups in this study [52]. Nevertheless, clonal *P. falciparum* population dominance is expected to be observed in regions with declining endemicity as a result of genetic drift caused by scale-up interventions [53]. In other words, in parasite populations where control efforts have left behind lineages that create clusters of highly related parasites, the persistence of a specific haplotype throughout the year is expected [53,54]. As a result, unrelated parasites are unlikely to coexist in the same mosquito blood meal [53].

In addition, the protective role of antibodies to malaria cannot be overemphasized, as studies have shown that the antibody response plays a vital role in the protection of pregnant women against PAM infection [55,56]. A sero-surveillance study in Thai-Myanmar at the Shoklo Malaria Research Centre reported a significant variation in the antibody profiles of *Pf*AMA_1_*, Pf*EBA175*, Pf*MSP_2_*,* and *Pf*VAR_2_CSA. Most controls’ antibody profiles for *Pf*MSP_3_ and *Pv*AMA_1_ remained low throughout gestation regardless of malaria exposure, whereas the antibody response varied more among the cases and the control group of pregnant women with and without infection [57]. This study is in line with a study in Yaoundé that instead looked at the antibody response of malaria vaccine candidates among children [58]. This confirms that during a malaria infection, antibody levels should rise as an indication of immune protection. Alistair et al [59] even demonstrated that babies born to women with PAM with high levels of VAR2CSA antibodies are protected against the vertical transmission of malaria and have a higher mean birth weight than those with low VAR2CSA antibody levels [59].

In contrast, studies have shown that HIV infection amplifies the effect of malaria, especially among pregnant women [39,43,44]. One of these effects is the dysregulation of immunity to malaria in mothers and even their children exposed in utero [12,60]. In Yaoundé, it was reported that IgG levels to CSP and TCC were lower in mothers with HIV, as were cord IgG levels to CSP, MSP1, and TCC in neonates born to mothers with HIV [12]. This was in line with a study in Kenya that reported that HIV infection is associated with a decrease in the magnitude and breadth of IgG responses to merozoite antigens, as well as a delay in the age-related acquisition of the IgG antibody response to schizont extract [9]. The study also revealed that children with HIV are less likely to be high responders to AMA1 [9]. This effect can be seen in children, as studies have reported that children exposed to HIV are more susceptible to infections than children not exposed to HIV [60]. However, there is still a lack of understanding of why children born to these women are more susceptible to infections [60]. To better understand why children exposed to HIV are more susceptibility to infections, such as malaria, studies evaluating the effect of HIV infection on the vertical transfer of mononuclear WBCs, as well as a longitudinal study monitoring the immune response of these children exposed over time, are needed.

Cytokines and chemokines are elevated in peripheral blood during an acute malaria infection and contribute to parasite clearance, but they are also likely to be responsible for many of the symptoms and pathological changes seen during malaria [61,62]. The balance of pro- and anti-inflammatory signals appears to play a significant role in determining the outcome of an infection (ie, whether it leads to protection or immunopathology) [62]. Eduard et al [63] reported that an imbalanced pro-inflammatory cytokine response may exacerbate infection severity. Among pregnant women in Cameroon, it was realized that placental malaria reduces maternal plasma levels of IL-17E, IL-27, and IL-28A, as well as neonatal plasma levels of IL-17E and IL-28A, which may aid in parasite clearance and increase child birth weight [14]. This is in line with a similar study carried out by Megnekou et al [64]. Nonetheless, Djontu an coworkers [65] showed that IL-28A, IL-27, and IL-17E levels are higher in cord plasma than in maternal peripheral plasma and that they correlate positively with increasing baby birth weight. This positive relationship between birth weight and both Th1- and Th2-promoting cytokines (IL-27, IL-28A) is consistent with the findings of Chêne et al [65], who found a link between low birth weight and lower levels of IFNγ and IL-5 in intervellous plasma samples from Beninese women. A study carried out in Abia State, Nigeria, showed that cytokine levels in the same woman's peripheral and placental blood may differ [66]. It was realized that IFNγ is significantly higher in peripheral blood than in placental blood. IL-4 and IL-10 levels were significantly lower in peripheral blood than in placental blood [66].

Among pregnant women with HIV, it was discovered that cytokine activity during pregnancy, as measured by plasma cytokine concentrations, varies with antiretroviral therapy and *P. falciparum* coinfection [67]. In Mozambique, it was reported that among adults with HIV, IL-8 and IFNγ-induced protein 10 (IP-10) play a complex role in inflammation during *P. falciparum* infection and a potential pathogenic role [67]. Another study reported that patients with HIV and cerebral malaria have lower median plasma levels of TNF-α and IL-10 than patients without HIV [68]. This goes to confirm that HIV affects the cytokine response to malaria infection. Nevertheless, information about the cytokine response to malaria among pregnant women with HIV is scanty. However, studies comparing the levels of cytokine response in pregnant women with HIV and with and without malaria, as well as pregnant women without HIV and with and without malaria, are required to better understand the role of cytokines in the pathogenesis of malaria.

### Future Directions

There is a need for research into the prevalence of malaria among pregnant women in Kumba, considering all trimesters. It is also necessary to conduct a study on *P. falciparum* resistance to SP-IPT as well as a longitudinal study on children born to mothers with HIV in order to understand why these children are more vulnerable to infections.

### Conclusion

The results of previous studies depict that HIV affects the prevalence of malaria among pregnant women irrespective of gravidity; malaria prevalence turns out to be much higher among pregnant women with than pregnant women without HIV. It was also revealed that more **P. falciparum* MSP1* alleles are associated with malaria infection among individuals with HIV and that genetic diversity is a function of malaria transmission in specific regions. In addition, HIV infection lowers the level of IgG response of antibodies to malaria infection for both mother and newborn babies. The likelihood of children with HIV to develop an immune response to AMA1 is low. HIV infection also alters the levels of the pro- and anti-inflammatory cytokine response, contributing to the lack of immune parasite clearance and parasite pathogenicity.

The findings of this work will be published in peer review journals and will be presented in seminars as well as conferences. Copies of the work will be submitted at the Pan-African University of Life and Earth Sciences Institute, University of Ibadan, Nigeria; the University of Buea; and the hospitals where the samples were collected. A copy will also be sent to the Union for African Population Studies.

## Abbreviations

AMA1: apical membrane antigen-1
ANC: antenatal care
CSP: circumsporozoite protein
EBA: erythrocyte-binding antigen
IFNγ: interferon gamma
Ig: immunoglobulin
IL: interleukin
IPT: intermittent preventive treatment
MSP: merozoite surface protein
PAM: pregnancy-associated malaria
PCR: polymerase chain reaction
*P. falciparum*: *Plasmodium falciparum*
RDT: rapid diagnostic test
SP: sulfadoxine-pyrimethamine
SWR: southwest region
TCC: terminal complement complex
TGF: transforming growth factor
Th cell: helper T cell
TNF-α: tumor necrosis factor alpha
Treg: regulatory T-cell
WBC: white blood cell

## Data Availability

The terms and conditions established by the local ethical committee will govern whether the data sets generated/analyzed during this study will be made public. However, upon reasonable request, they will be made available to the corresponding authors.
